# Characterization of *Stackebrandtia nassauensis* GH 20 Beta-Hexosaminidase, a Versatile Biocatalyst for Chitobiose Degradation

**DOI:** 10.3390/ijms20051243

**Published:** 2019-03-12

**Authors:** Meng Wang, Feng Zheng, Ting Wang, Yong-Mei Lyu, Matthew G. Alteen, Zhi-Peng Cai, Zhong-Li Cui, Li Liu, Josef Voglmeir

**Affiliations:** 1 Glycomics and Glycan Bioengineering Research Center (GGBRC), College of Food Science and Technology, Nanjing Agricultural University, Nanjing 210095, China; 2016208028@njau.edu.cn (M.W.); 2016108004@njau.edu.cn (F.Z.); wangting@njau.edu.cn (T.W.); 2016208021@njau.edu.cn (Y.-M.L.); caizhipeng@njau.edu.cn (Z.-P.C.); 2 Department of Chemistry, Simon Fraser University, Burnaby, BC V5A 1S6, Canada; malteen@sfu.ca; 3 College of Life Sciences, Nanjing Agricultural University, Nanjing 210095, China; czl@njau.edu.cn

**Keywords:** β-*N*-acetylhexosaminidases, Glycan degradation, exochitinase, GH20 family, *Stackebrandtia*

## Abstract

An unstudied β-*N*-acetylhexosaminidase (SnHex) from the soil bacterium *Stackebrandtia nassauensis* was successfully cloned and subsequently expressed as a soluble protein in *Escherichia coli*. Activity tests and the biochemical characterization of the purified protein revealed an optimum pH of 6.0 and a robust thermal stability at 50 °C within 24 h. The addition of urea (1 M) or sodium dodecyl sulfate (1% *w*/*v*) reduced the activity of the enzyme by 44% and 58%, respectively, whereas the addition of divalent metal ions had no effect on the enzymatic activity. PUGNAc (*O*-(2-acetamido-2-deoxy-D-glucopyranosylidene)amino-*N*-phenylcarbamate) strongly inhibited the enzyme in sub-micromolar concentrations. The β-*N*-acetylhexosaminidase was able to hydrolyze β1,2-linked, β1,3-linked, β1,4-linked, and β1,6-linked GlcNAc residues from the non-reducing end of various tested glycan standards, including bisecting GlcNAc from one of the tested hybrid-type N-glycan substrates. A mutational study revealed that the amino acids D306 and E307 bear the catalytically relevant side acid/base side chains. When coupled with a chitinase, the β-*N*-acetylhexosaminidase was able to generate GlcNAc directly from colloidal chitin, which showed the potential of this enzyme for biotechnological applications.

## 1. Introduction

β-*N*-acetylhexosaminidases (EC 3.2.1.52) are a class of enzymes that hydrolyze the outermost β-linked *N*-acetylglucosamine (GlcNAc) and *N*-acetylgalactosamine (GalNAc) residues from glycoconjugates [1]. These enzymes are widely distributed in eukaryotes [2,3,4,5], bacteria [6,7,8,9], archaea [10], and viruses [11]. β-*N*-acetylhexosaminidases are essential for degrading carbohydrates such as complex glycans [12] or chitooligosaccharides [13], and are described to be important for recycling cell metabolites [14,15] and plant development [16]. Structure-wise, β-*N*-acetylhexosaminidases can be divided into CAZy glycoside hydrolase families GH3, GH20, GH84, and GH116, of which the family GH20 is predominantly studied. Commercial GH20 β-*N*-acetylhexosaminidases are currently either isolated from plant or fungal hosts such as *Canavalia ensiformis* (the common jack bean) [17] or *Aspergillus oryzae* (koji) [18,19], or derived recombinantly from bacteria such as *Streptomyces plicatus* [20] or *Streptococcus pneumonia* [21]. These groups of enzymes could potentially be applied in enzymatic cascades for the generation of GlcNAc from chitinous by-products from the industrial fermentation of filamentous fungi (i.e., *Aspergillus* sp. or *Rhizopus* sp.) or from crustaceans (i.e., crabs or shrimps) in the future. Although no large-scale applications of GlcNAc are described ( as starting material for the chemical or enzymatic synthesis of bioethanol or other bulk chemicals), the investigation of alternative carbohydrate resources besides glucose or fructose is required. However, to further these biotechnological attempts, highly active and robust β-*N*-acetylhexosaminidase variants, which are suitable for large scale fermentations would be highly desirable. To expand the current pool of β-*N*-acetylhexosaminidases, we cloned an unstudied candidate gene derived from the soil bacterium *Stackebrandtia nassauensis*. This bacterium was first isolated in the Bahamas in 2004 [22] and, since the genome sequence is accessible [23], *S. nassauensis* has been investigated for its unique type of bioactive lantipeptides (now known as stackepeptins) [24], novel imine reductases [25], and epoxide hydrolases [26]. In this scenario, we explore the biochemical properties and substrate specificities of this *S. nassauensis* β-*N*-acetylhexosaminidase (henceforth referred to as SnHex).

## 2. Results

### 2.1. Gene Cloning of SnHex

**Figure 1:**
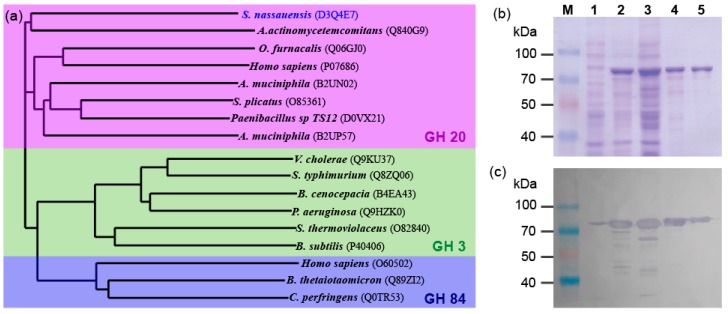
(**a**) Schematic representation of the phylogenetic relationship of functionally characterized β-*N*-acetylhexosaminidases (UniProt identifiers shown in parentheses). (**b**) SDS-PAGE analysis and (**c**) Western blotting (using anti-polyHistidine antibody) of recombinant SnHex at various stages of expression and purification. M—protein marker, 1—cell pellet before induction, 2—cell pellet after induction with IPTG, 3—supernatant after cell lysis, 4—SnHex purified by the Ni-NTA resin, 5—SnHex after a second purification step using anion exchange chromatography.

An open reading frame encoding a putative β-*N*-acetylhexosaminidase candidate gene was successfully amplified by a polymerase chain reaction (Figure S1). The 1578 base pair fragment was sequenced and showed no mutations when compared with the reference gene sequence from GeneBank accession NC_013947. The encoding 526 amino acids showed the highest similarity with characterized GH20 enzyme family members, and shared 31% identity with β-*N*-acetylhexosaminidases from *Paenibacillus* sp. TS12 (UniProt ID D0VX21) [27], 38% identity with *S. plicatus* (UniProt ID O85361) [28], and 24% with both *A. actinomycetemcomitans* (UniProt ID Q840G9) [29] and *Ostrinia furnacalis* (UniProt ID: Q06GJ0) [30] homologs. A phylogenetic comparison of previously characterized β-*N*-acetylhexosaminidases belonging to known β-*N*-acetylhexosaminidase CAZy families clearly confirmed that SnHex belongs to the GH20 family (Figure 1a).

### 2.2. Protein Expression and Purification

The recombinant form of SnHex could be successfully expressed in soluble form (Figure 1b). A hexahistidine tag fused to the C-terminus further allowed the visualization of SnHex by Western blotting (Figure 1c). The purified sample migrated as a dominant protein band with a molecular weight between 70 kDa and 100 kDa, which was bigger than the expected mass of 58 kDa. However, MALDI-ToF mass spectrometric analysis of the purified protein sample showed *m*/*z* values of 29 kDa (*z* = 2) and 57.8 kDa (*z* = 1), which is in good agreement with the expected mass value for SnHex (Figure S2). The protein concentration of the purified SnHex was determined to be 1.46 ± 0.09 mg/mL. The specific activity was calculated to be 0.62 ± 0.02 U per µg of protein.

### 2.3. Biochemical Characterization

**Figure 2:**
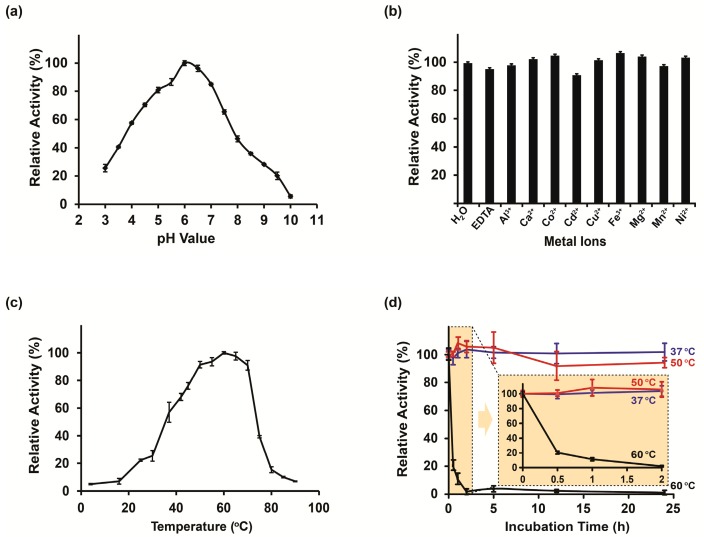
Biochemical characterization. (**a**) pH-dependency of recombinant SnHex. (**b**) Impact of metal ions and EDTA on the enzymatic activity of SnHex. (**c**) Relative activity of SnHex at various incubation temperatures using 10 min enzymatic reactions. (**d**) Thermal stability of SnHex using multiple pre-incubation times and temperatures. The orange cutout is an enlarged view of the stability values between 0 h and 2 h.

The activity of SnHex was tested in a colorimetric assay using *p*NP-β-GlcNAc as the substrate by measuring the absorbance of the released *p*-nitrophenol used a spectrophotometer at 405 nm. SnHex showed good enzymatic activity in the pH range between 4.0 and 8.0, with the highest activities measured at a pH of 6.0 (Figure 2a). The addition of metal ions (Al^3+^, Ca^2+^, Co^2+^, Cd^2+^, Cu^2+^, Fe^3+^, Mg^2+^, Mn^2+^, and Ni^2+^ in their chloride forms) or EDTA did not reduce the activity of the enzyme when added at a final concentration of 2 mM (pH 6.0, 37 °C reaction temperature). These data suggest that the catalytic mechanism of the enzyme is not metal ion-dependent (Figure 2b). When incubating SnHex for short periods of time (10 min), the highest activities were measured at 60 °C (Figure 2c). SnHex was reasonably stable for at least 24 h at 37 °C and 50 °C, while pre-incubation of the enzyme solution at 60 °C led to its complete inactivation within 1 h (Figure 2d). The non-specific β-*N*-acetylhexosaminidase inhibitor PUGNAc strongly inhibited SnHex activity when used in sub-micromolar concentrations (Figure S3). Furthermore, the activity of SnHex was not significantly influenced when adding various concentrations of 2-mercaptoethanol, imidazole, iodoacetamide, or Triton X-100 to the reaction mixture (Table S1). The addition of urea (1M) or SDS (0.1%) reduced activity by approximately half (Table S1). The determined V_max_, *K*_M_, and k_cat_ were similar when using *p*NP-β-GlcNAc and *p*NP-β-GlcNAc as substrates, with a slight preference for catalyzing the hydrolysis of *p*NP-β-GlcNAc (Table 1).

### 2.4. Substrate Specificity

**Figure 3:**
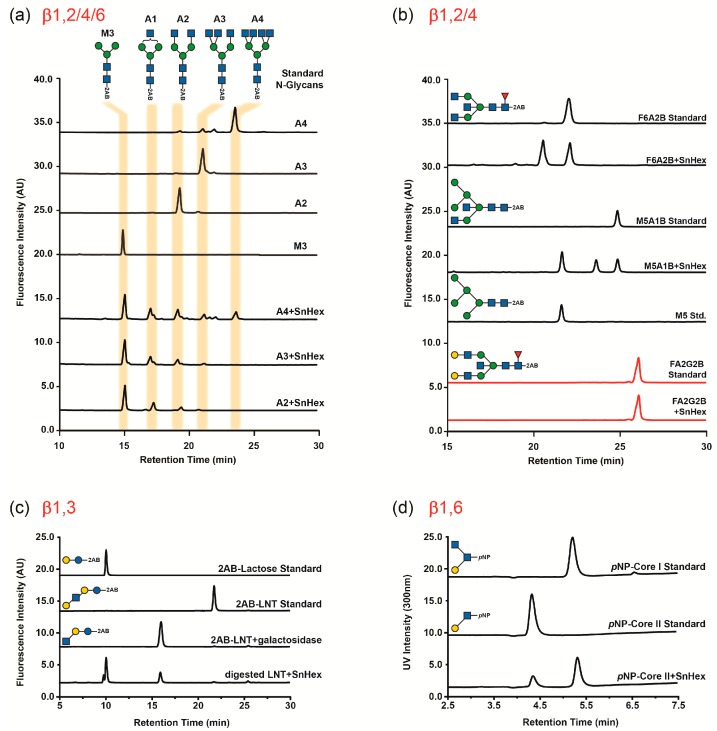
HPLC traces showing hydrolysis of various terminal GlcNAc residues from glycan substrates by SnHex. A detailed depiction of the glycan structures can be found in the supplementary information (Table S2).

The selectivity of SnHex was tested using a panel of *p*-nitrophenyl glycosides, of which only pNP-β-GlcNAc and pNP-β-GalNAc were hydrolyzed (Figure S4). The enzyme was also able to hydrolyze terminally linked β-1,2, β-1,4, and β-1,6 GlcNAc residues from various di-antennary, tri-antennary, and tetra-antennary *N*-glycan standards (Figure 3a). The *N*-glycan standards F6A2B and M5A1B (Table S2), which bear bisecting GlcNAc moieties (β-1,4-linked to the β-linked core mannose), were hydrolyzed less efficiently by SnHex, and no GlcNAc hydrolysis was detected using the *N*-glycan standard F6A2BG2 even after prolonged incubations with SnHex (Figure 3b). The enzyme could also release β-1,3-linked GlcNAc from the milk oligosaccharide LNT after β-galactosidase treatment of the sample (Figure 3c). Furthermore, SnHex was able to release terminal β-1,6 linked GlcNAc from a core-II glycan standard, which yields the core-I epitope as a reaction product (Figure 3d). Much to our surprise, the enzyme was also able to hydrolyze serine-linked β-GlcNAc from a glycopeptide (Figure S5).

### 2.5. Mutational Analysis

Protein sequence alignment with the functionally characterized human β-*N*-acetylhexosaminidase D (UniProt ID Q8WVB3) suggested that the two catalytic amino acids of SnHex are D306 and E307 (Figure S6). Therefore, these amino acids were targeted by site-directed mutagenesis generating the single amino acid mutants D306A, D306E, D306N, E307A, E307D, E307Q, and the double amino acid mutant D306E/E307D. When using *p*NP-GlcNAc as a substrate, mutants D306A and D306N completely lost all catalytic activity while other mutations resulted in a reduction of the enzymatic activity when compared to the wild-type enzyme (Table 2). None of the mutant SnHex enzyme variants could release GlcNAc residues when testing the activity towards the biantennary *N*-glycan standard A2 (Table 2 and Figures S7 and S8).

### 2.6. GlcNAc Generation from Colloidal Chitin

**Figure 4:**
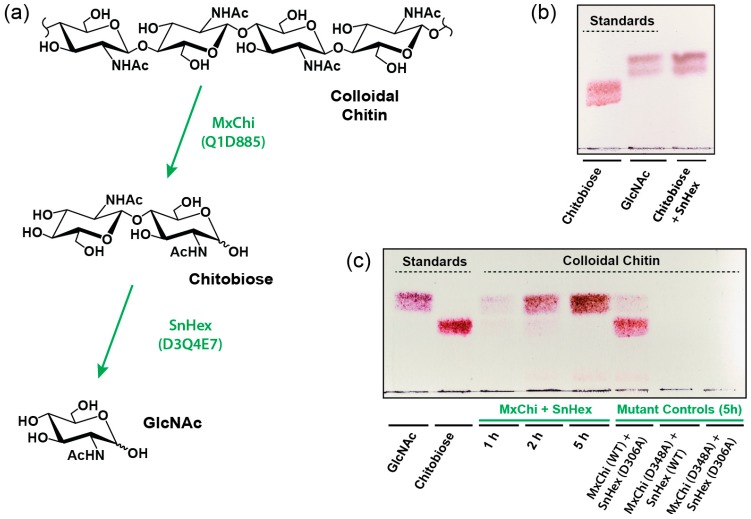
Enzymatic degradation of colloidal chitin. (**a**) Schematic overview of the action of MxChi and SnHex on colloidal chitin and chitobiose substrates. (**b**) TLC analysis using chitobiose as a substrate for the enzymatic hydrolysis to GlcNAc by SnHex. (**c**) TLC analysis using colloidal chitin as the substrate after incubation with both MxChi and SnHex, which yields the enzymatic hydrolysis product GlcNAc. The two bands in each analysis plate results from the α-anomers and β-anomers of chitobiose and GlcNAc. TLCs were developed with a mobile phase containing n-butanol:methanol:water at a ratio of 5:3:2 and were stained with DPA.

In this work, we also wanted to demonstrate that SnHex can be used in an enzymatic cascade, in which GlcNAc can be produced enzymatically from chitin. Therefore, we decided to generate chitobiose from chitin using the previously unstudied chitinase MxChi from *Myxococcus xanthus* (UniProt identifier Q1D885, Figure S9), while adding SnHex to the same reaction mixture to generate GlcNAc from chitobiose (Figure 4a). The efficiency of SnHex to hydrolyze chitobiose was successfully evaluated in a separate overnight reaction (Figure 4b). The role of each enzyme in the hydrolysis of colloidal chitin was further investigated by using combinations of wild-type and inactivated mutant enzymes of MxChi and SnHex, which shows that both enzymes are required for the effective production of GlcNAc (Figure 4c).

## 3. Discussion

### 3.1. Enzyme Characterization

SnHex was successfully expressed in a recombinant form in *E. coli*. SnHex showed the highest enzymatic activity of 35 unstudied bacterial β-*N*-acetylhexosaminidase candidates tested in initial activity screens (Table S3). The enzyme was purified as a single protein band, which migrated between 70–100 kDa. This is much higher than the expected molecular weight of 58 kDa. Surprised by this discrepancy, we performed a mass spectrometric analysis of the purified protein, which confirmed the expected molecular weight of 58 kDa for SnHex (Figure S2). The rather unusual migration behavior on the SDS-PAGE gel may indicate that SnHex is not fully denatured upon heating in the SDS-PAGE loading buffer, which is a phenomenon that results in the shift of protein bands from their expected molecular weight. This was investigated for various proteins by Rath et al. [31]. Robust expression results were achieved using *E. coli* BL21 (DE3) lacZ^−^ as the expression host. Furthermore, the enzymatic activity of SnHex was unambiguously demonstrated using various substrates and analytical methods.

The pH optimum of SnHex was comparable with other characterized β-*N*-acetylhexosaminidases, which were generally described to be in the range between pH 5.5 and pH 6.5 [6,32,33,34]. The optimum temperature of SnHex was determined to be 60 °C when incubated for a short period of time (10-min), but the enzyme was very unstable at this temperature when it is exposed for more than 30 min (Figure 2d). However, extended incubations at 50 °C or below did not result in a significant loss of activity. Most bacterial β-*N*-acetylhexosaminidases described so far have a rather low thermal stability [6,8,35]. The properties of SnHex seem to be similar to a β-*N*-acetylhexosaminidase isolated from the thermophilic bacterium *Bacillus stearothermophilus* [33]. This enzyme was described to have a rather high temperature maximum at short time incubations (75 °C), but was only moderately stable above 60 °C, losing 40% of its initial activity when exposed for 10 min at a temperature of 70 °C. Furthermore, an archaeal bifunctional glucosidase/*N*-acetyl-β-glucosaminidase isolated from *Sulfolobus solfataricus* was described to have excellent thermal stability at 65 °C [36]. The addition of EDTA did not inhibit the activity of SnHex, which confirms that metal ions are not required in the catalytic mechanism of the enzyme. Even though some other characterized β-*N*-acetylhexosaminidases were reported to be sensitive to various metal ions [33,37,38], no effect was detected on the activity of SnHex. In addition, 2-mercaptoethanol did not significantly influence the enzyme’s activity, which suggests that the oxidized form of cysteine residues is not essential for the activity of the enzyme. Using the SignalP 4.0 online tool [39], it was predicted that SnHex contains an *N*-terminal signal sequence. This indicates that this enzyme is secreted by *S. nassauensis*. Taken together, SnHex can be active in a relatively broad range of physiological conditions and is, therefore, potentially suitable for multi-enzyme applications.

The kinetic analysis showed that the measured *K*_M_ values of 2.47 mM for *p*NP-β-GlcNAc and 3.29 mM for *p*NP-β-GalNAc were slightly higher when compared to values described for other characterized β-*N*-acetylhexosaminidases, which were reported in the range between 0.12 mM and 0.51 mM for *p*NP-β-GlcNAc, and between 0.11 mM and 1.0 mM *for p*NP-β-GalNAc [6,27,40,41,42]. Higher *K*_M_ values were described for the β-*N*-acetylhexosaminidases from *Aeromonas hydrophila* (8.6 mM for *p*NP-β-GlcNAc and 11.1 for *p*NP-GalNAc) [32]. SnHex appears to show a slight preference for β-GlcNAc substrates over β-GalNAc, which is in agreement with the substrate preference of most GH20 β-*N*-acetylhexosaminidases. The catalytic efficiency of SnHex (k_cat_/*K*_M_) is approximately two-fold higher for *p*NP-β-GlcNAc *vs p*NP-β-GalNAc. Consistent with the established mechanism of GH20 enzymes, SnHex did not show activity towards α-linked substrates or substrates lacking an *N*-acetamido group at the two-position of the carbohydrate (Figure S4).

### 3.2. Glycan Substrate Promiscuity

By testing various glycan standards, it was established that SnHex was able to hydrolyze β-1,2, β-1,3, β-1,4, and β-1,6 linked GlcNAc moieties. This activity is more promiscuous than that described for other bacterial β-*N*-acetylhexosaminidases. For example, the characterized β-*N*-acetylhexosaminidases from *Diplococcus pneumonia* could not cleave GlcNAcβ1,2 Man linkages using GlcNAcβ1,6(GlcNAcβ1,2)Man as a substrate even though it hydrolyzed this motif using GlcNAcβ1,4(GlcNAcβ1,2)Man as a substrate [21]. None of the characterized β-*N*-acetylhexosaminidase isoforms from *Akkermansia muciniphila* were able to hydrolyze any of the terminally branched GlcNAc moieties from tri-antennary and tetra-antennary *N*-glycan standard substrates [6]. In this respect, SnHex seems to be as promiscuous as eukaryotic β-*N*-acetylhexosaminidases, which were also described to release a broad range of β-linked GlcNAc moieties [17]. However, despite demonstrating the release of β-1,2, β-1,3, β-1,4, and β-1,6 linked GlcNAc residues, a generalization on the substrate specificity could not be clearly revealed. This was exemplified by screening two-to-one bisecting *N*-glycan standards (F6A2B and M5A1B, Figure 3b), of which only the further one was an acceptor substrate for SnHex. A further generalization of the substrate specificity will require a detailed structural study of SnHex.

### 3.3. Molecular Mechanism

**Figure 5:**
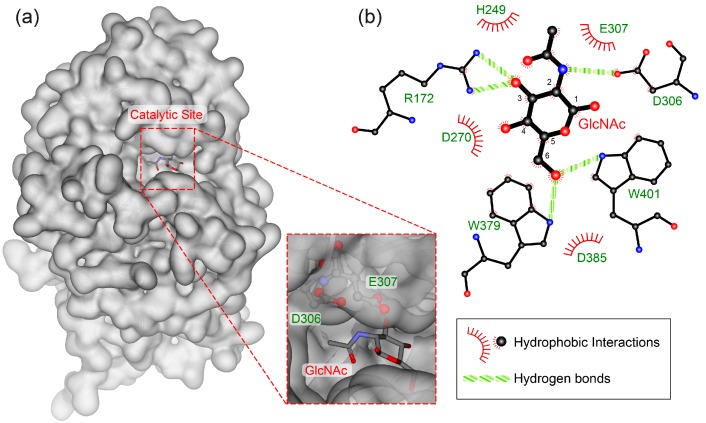
(**a**) Three-dimensional homology model of SnHex based on the structure of the highly homologous β-*N*-acetylhexosaminidase from *Streptomyces plicatus* complexed with GlcNAc (PDB code 1M01) with the amino acids D306 and E307 located at the catalytic site. (**b**) Two-dimensional illustration of the substrate-ligand interactions in the active site of SnHex.

The catalytic mechanism of GH20 β-*N*-acetylhexosaminidases has been studied extensively. Enzymes in this family operate using a substrate-assisted catalytic mechanism in which the *N*-acetamido group acts as a nucleophile to displace the anomeric leaving group, which results in a bicyclic oxazoline intermediate. A conserved Asp-Glu amino acid pair act as catalytic acid-base residues to facilitate the departure of the leaving group and polarization of the *N*-acetamido group [2,9,43,44]. The mutation of these two residues (D306 and E307) resulted in complete loss of activity when using an *N*-glycan standard (A2, Table S2) as a substrate. In the case of using *p*NP-β-GlcNAc as a substrate, only the mutation of D306 resulted in the complete loss of SnHex activity, whereas residual enzymatic activities could still be determined for all E307 mutants. This can be rationalized by the low pK_a_ of the phenolic leaving group, which therefore does not entirely depend on the assistance of E307 for the hydrolysis reaction [2]. A protein alignment of SnHex with characterized GH20 β-*N*-acetylhexosaminidases also revealed the same catalytically-relevant motif in SnHex (Figure S6), and the exchange of these carboxylic acid residues with other amino acids reduced or abolished the catalytic activity of the enzyme (Table 2, Figures S7 and S8). A homology model of SnHex based on the crystal structure of *Streptomyces plicatus* β-*N*-acetylhexosaminidase showed that the catalytic residues D306 and E307 are part of the active site and in the proximity of the complexed GlcNAc moiety (Figure 5).

## 4. Materials and Methods

### 4.1. General

Enzymes for DNA manipulation (Nco *I*, Xho *I* & T4 ligase) were purchased from Thermo Fisher Scientific (Shanghai, China). Primestar HS DNA polymerase was purchased from Takara (Dalian, China). DNA Gel Purification and Plasmid Extraction kits were purchased from Axygen (Beijing, China). The pET-30a(+) expression vector was purchased from Novagen (Madison, Wisconsin, USA). *Stackebrandtia nassauensis* strain DSM 44728 was obtained from the German Collection of Microorganisms and Cell Cultures (DSMZ). *Escherichia coli* Mach1 T1 cells (Life Technologies, China) were used for plasmid amplification and manipulation procedures. *E. coli* strain BL21(DE3) (Invitrogen) was used to generate a knockout strain without endogenous β-galactosidase activity (*E. coli* BL21(DE3) lacZ-) using the λ-Red recombinase system [45,46]. The lacz knockout primer pair 5’-TATGTTGTGTGAAATTGTGAGCGGATAACAATTTCACACAGGAAACAGCTGT GTAGGCTGGAGCTGCTTC-3’ and 5’-ATGGATTTCCTTACGCGAAATACGGGCAGACATGGCC TGCCCGGTTATTAATGGGAATTAGCCATGGTCC-3’ was used for the genomic excision of the LacZ-gene, which resulted in colonies showing no β-galactosidase activity upon blue/white screening. DNA primers were obtained from GenScript (Nanjing, China). TRIzol was purchased from Invitrogen (Shanghai, China). Bulk chemicals used in this study were purchased from various local chemical suppliers.

### 4.2. Gene Amplification, Construction of the Expression Vector, and Mutant Generation

Genomic DNA was isolated from a freeze-dried cell pellet of *Stackebrandtia nassauensis* as previously described for another bacterial strain [47] based on the method described by Mahuku [48]. The DNA primers for amplifying the putative β-*N*-acetylhexosaminidase gene were designed using the genomic data provided by the Pathosystems Resource Integration Center (PATRIC) [49] (sn.ggbrc.com) and consists of the following base pairs: 5′-CATGCCATGGTGGCCGATCCG TCCCAC-3′ (forward) and 5′-CCGCTCGAGTTACAAAGATGAAAAGCCCG-3′ (reverse). The gene amplification was carried out using 35 PCR cycles consisting of denaturation at 95 °C for 10 s, annealing at 55 °С for 30 s, and elongation at 72 °С for 1 min. The PCR fragments were purified on an agarose gel, digested with the restriction endonucleases *Nco* I and *Xho* I, and subsequently ligated into a pET-30a expression vector. By using these restriction sites, the open reading frame of the final vector construct also carries an N-terminal Histag/thrombin/S-tag configuration followed by an enterokinase cleavage site. The ligation mixtures were transformed into *E. coli* Mach1 T1 competent cells and were selected on lysogeny broth (LB) agar containing 50 µg/mL kanamycin. A clone containing the expected plasmid construct was stored at −80 °C and used for further experiments. Active site mutants were generated using the standard protocol for QuikChange Site-Directed Mutagenesis (Stratagene) with the primer pairs listed in Table S4. Plasmid extraction, DNA ligation, purification, and transformation procedures were carried out using standard protocols.

### 4.3. Protein Expression and Purification

*E. coli* BL21 (DE3) (lacZ^−^) cells transformed with the SnHex/pET-30a construct were grown overnight at 37 °C in 5 mL of LB medium containing 50 μg/mL of kanamycin with 200 rpm of shaking. One mL of the culture was then transferred into a 2 L baffled Erlenmeyer flask containing 400 mL of LB medium and was shaken at 37 °C until the optical density of the cells at 600 nm (OD_600_) reached a value of approximately 0.5. Then, the temperature of the fermentation broth was lowered to 18 °C, and the protein expression was induced by adding 1 mM IPTG. After a further 16 h of shaking, the induced cells were harvested by centrifugation (15 min at 5000× *g*), re-suspended in lysis buffer (50 mM Tris, 100 mM NaCl, 1% (*w*/*v*) Triton X-100, adjusted to pH 8.0 with HCl), and lysed by sonication (40 on/off cycles with 20 μm probe amplitude for 15 s). After centrifugation at 14,000× *g* for 20 min, the supernatant of the cell lysate was collected and loaded onto a Ni^2+^-NTA Sefinose resin (1 mL bed volume, BBI Life Science, Shanghai, China). Fractions showing the highest UV absorbance at 280 nm were pooled and desalted on a disposable PD-10 cartridge (GE Healthcare) using Tris/HCl buffer (20 mM, pH 8.0) as an elution medium. The desalted eluent was then loaded onto a pre-packed anion exchange cartridge (Q FF, 1 mL, GE Healthcare), which was equilibrated with Tris/HCl buffer (20 mM, pH 8.0). The target protein was eluted over 30-min using a linear NaCl gradient (0 to 1M) with a 1 mL/min flow rate. Active SnHex eluted at 16.8 min, which corresponds to 560 mM NaCl in 20 mM Tris/HCl buffer (pH 8.0).

The expression level and purity of SnHex during protein expression, cell lysis, and at the various protein purification steps were monitored by SDS-PAGE and visualized using Coomassie brilliant blue G-250. For Western blotting, proteins separated on an acrylamide gel were transferred to nitrocellulose using a semi-dry blotting apparatus. After blocking the protein binding of the membrane with 0.5% (*w*/*v*) bovine serum albumin, it was incubated with mouse anti-polyHistidine antibody (1:10,000) and, after washing, incubated with an alkaline phosphatase conjugated goat anti-mouse IgG antibody (1:10,000). After washing, the blots were stained using the BCIP/NBT reagent as phosphatase substrate.

The purified protein fractions of the highest purity were pooled and stored in 30% glycerol (*w*/*v*) at −80 °C for further experiments. The protein concentration of this pool was measured using a Bradford protein quantification kit (Sangon Biotech, Shanghai, China).

### 4.4. Activity Assays and Biochemical Characterization

The activity of SnHex was measured by monitoring the amounts of released *p*-nitrophenol (*p*NP) from the *p*NP-β-GlcNAc photometrically at a wavelength of 405 nm. These measurements were typically performed in 25 µL mixtures of 50 mM phosphate-citric acid buffer (‘McIlvaine buffer’, pH 6.0) containing 1 mM of *p*NP-β-GlcNAc as substrate and 0.1 U of SnHex. For quantitative analysis, reactions were quenched after typically 10 min of reaction time by adding 25 µL of Na_2_CO_3_ solution (1 M) prior to measurements. The metal ion dependency was measured by adding Al^3+^, Ca^2+^, Cd^2+^, Co^2+^, Cu^2+^, Fe^3+^, Mg^2+^, Mn^2+^, and Ni^2+^ ions as chlorides at a final concentration of 2 mM. The substrate specificity was measured by replacing *p*NP-β-GlcNAc with other *p*NP-glycosides. The effect of additives, denaturants or PUGNAc was measured by adding these compounds to the reaction mixture at various concentrations. All enzymatic measurements were performed using three technical replicates. The kinetic parameters of SnHex were determined with various concentrations of *p*NP-β-GlcNAc and *p*NP-β-GalNAc (final assay concentrations of 0.01, 0.02, 0.05, 0.1, 0.2, 0.5, 1.0, 2.0, 5.0, and 10 mM) as substrates. *K_M_* and *V_max_* were calculated using a non-linear regression model, according to the method previously described [50]. One unit (U) of SnHex activity was defined as the amount of enzyme necessary to release 1 μmol of *p*-nitrophenol from *p*NP-β-GlcNAc within 1 min at 37 °C.

### 4.5. Glycan Analysis

In addition, 2.5 μL of 20 mM chitobiose was hydrolyzed by 1.4 U of SnHex in 50 mM of MES buffer (pH 6.0) at 37 °C in a 16-h reaction. Seventy-five μL of colloidal chitin gel (0.94 mg, 0% degree of acetylation) were treated with 15 μg of MxChi and 9.3 U (15 μg) of SnHex at 37 °C in MES buffer (pH 6.0). The catalytically inactive mutant variants of SnHex D306A and MxChi D348A were used in control experiments. Reaction mixtures were separated after 1 h, 2 h, and 5 h reaction times using thin layer chromatography (silica gel F 254 plates, Merck), by using the solvent mixture *n*-butanol:methanol:water at a ratio of 5:3:2 as mobile phase and DPA staining for visualization.

Underivatized glycan standards were obtained from Prozyme (Hayward, California, USA) and labeled with 2-aminobenzamide using the method described by Du et al. [51]. In general, 1 pmol of the respective *N*-glycan standard were treated by approximately 0.6 U of SnHex in 20 mM MES buffer (pH 6.0, final volume 10 μL). In the case of bisecting *N*-glycan standard substrates, 3 U of SnHex were used. Reactions were usually incubated for 30 min at 37 °C. In the case of bisecting *N*-glycans, the reaction time was increased to 16 h. Reactions were quenched by heating the samples to 95 °C for 5 min, and the resulting precipitate was removed by centrifugation (13,000 rpm, 10 min). Ten μL of the clear supernatant were then mixed with 40 μL of acetonitrile and used for UPLC analysis, by using the previously described method by Wang et al. [52].

To test the activity of SnHex on a glycopeptide, a TAMRA-labeled CKII oligopeptide (TAMRA-YPGGSTPVSSANMM) was obtained commercially (Biomatik, Cambridge, ON, Canada) and was glycosylated chemoenzymatically using recombinantly-expressed *O*-GlcNAc transferase [2]. The oligopeptide (1 mM) was incubated with UDP-GlcNAc (5 mM), OGT (1 μM), and 2U shrimp alkaline phosphatase (New England Biolabs, Whitby, ON, Canada) in PBS at a pH of 7.2 containing 12.5 mM MgCl_2_. The reaction was incubated at 37 °C for 4 h, terminated by heating at 95 °C for 10 min, and centrifuged at 13,000× *g*. The supernatant was purified on an Agilent 1200 series HPLC using an Agilent XDB-C18 Eclipse reversed-phase column (9.4 × 250 mm, 5 μ particle size). The glycosylated CKII product was eluted using an H_2_O:CH_3_CN mobile phase (20% to 50% CH_3_CN over 20 min, 2mL/min flow rate, and UV detection at 570 nm) and was lyophilized to yield up to 1 mg glycopeptide (retention time 12.1 min, HRMS (ESI^+^) [M + 2H]^2+^ calculated *m*/*z* 1007.4158 Da, found 1007.4156 Da).

To evaluate SnHex hydrolysis activity on this substrate, 0.5 μL of the CKII-glycopeptide (20 μM concentration) was treated with 1 U of SnHex at 37 °C for 3 h. After quenching the reaction mixture by heating for 3 min to 95 °C, an aliquot of 1 μL was mixed with 1 uL of DHB (2,5-dihydroxybenzoic acid) matrix solution (consisting of 5 mg of DHB in 1 mL of 30% aqueous acetonitrile) and subjected to MALDI-TOF MS analysis (Bruker Autoflex). The analysis of the enzymatic hydrolysis of the *p*NP-core-II substrate was performed as previously described by Wang et al. [53].

### 4.6. Homology Modeling Phylogenetic Analysis

Comparative two-dimensional and three-dimensional protein models of SnHex were generated using the three-dimensional structure of the *Streptomyces plicatus* complexed with GlcNAc (PDB code 1M01), by utilizing LigPlot Plus (Version 1.4.) [54] and the MODELLER homology software (Version 9.17) [55]. The three-dimensional image of SnHEX was generated using Accelrys Discovery Studio Visualizer (Version 4.0). The phylogenetic relationship between β-*N*-acetylhexosaminidases and the sequence alignments were generated using the MUSCLE and PhyML online tools provided by Deereper et al. [56].

## 5. Conclusions

In this work, an unstudied β-*N*-acetylhexosaminidase was cloned successfully from the soil bacterium *S. nassauensis*. The enzyme could be recombinantly expressed and biochemically characterized. The reasonable thermal stability and the relative broad substrate promiscuity are promising features of this enzyme for further evaluation of this enzyme in analytical applications, such as exoglycosidase treatments of carbohydrate samples, or the enzymatic degradation of chitinous materials in biotechnological applications.

## Figures and Tables

**Table 1:** Kinetic parameters of recombinant SnHex.

*p*NP-β-GlcNAc				*p*NP-β-GalNAc			
V_max_ (mM·min^−1^·mg^−1^)	*K*_M_ (mM)	k_cat_ (min^−1^)	k_cat_/*K*_M_ (min^−1^mM^−1^)	V_max_ (mM·min^−1^·mg^−1^)	*K*_M_ (mM)	k_cat_ (min^−1^)	k_cat_/*K*_M_ (min^−1^mM^−1^)
0.057 ± 0.004	2.47 ± 0.05	55.05 ± 3.69	22.3 ± 2.0	0.044 ± 0.003	3.29 ± 0.32	42.72 ± 2.86	13.0 ± 2.3

**Table 2:** Activity of wild-type and mutant variants of SnHex using *p*NP-β-GlcNAc and the A2 *N*-glycan standard as substrates. ND: no detection of any SnHex enzyme activity.

SnHex Variant	Relative Activity (%)	
	*p*NP-β-GlcNAc	A2 *N*-Glycan Standard
**Wild-Type**	100 ± 2.1	100
**D306A**	ND	ND
**D306E**	4.2 ± 0.5	ND
**D306N**	ND	ND
**E307A**	2.0 ± 0.2	ND
**E307D**	8.6 ± 0.2	ND
**E307Q**	1.5 ± 0.2	ND
**D306E/E307D**	2.7 ± 0.3	ND

## Supplementary Materials

Supplementary materials can be found at https://www.mdpi.com/1422-0067/20/5/1243/s1.

## Abbreviations

BCIP/NBT	5-Bromo-4-chloro-3’-indolyphosphate/nitro-blue tetrazolium
CAZy	Carbohydrate-active enzymes database
dNTP	Deoxy-ribonucleotide triphosphate
DPA	Diphenylamine-aniline-phosphoric acid
DHB	2,5-Dihydroxybenzoic acid
EDTA	Ethylenediaminetetraacetic acid
ESI^+^	Electron spray ionization positive mode
HPLC	High-performance liquid chromatography
HRMS	High-resolution mass spectrometry
GalNAc	*N*-acetyl-D-galactosamine
GlcNAc	*N*-acetyl-D-glucosamine
IPTG	Isopropyl-β-D-thiogalactopyranoside
LB	Lysogeny broth
LNT	Lacto-*N*-tetraose
MALDI-TOF MS	Matrix-assisted laser desorption/ ionization time of flight mass spectrometry
MES	4-Morpholineethanesulfonic acid hydrate
OD	Optical density
OGT	*O*-GlcNAc transferase
PAGE	Polyacrylamide gel electrophoresis
PCR	Polymerase chain reaction
*p*NP	*para*-Nitrophenol
PUGNAc	*O*-(2-acetamido-2-deoxy-D-glucopyranosylidene)amino-*N*-phenylcarbamate
SDS	Sodium dodecyl sulfate
TAMRA	5-Carboxytetramethylrhodamine
UPLC	Ultra high-performance liquid chromatography
