# Supplementary material for: Characterization of Stackebrandtia nassauensis GH 20 Beta-Hexosaminidase, a Versatile Biocatalyst for Chitobiose Degradation
Source: Int J Mol Sci. 2019 Mar 12;20(5):1243. doi: 10.3390/ijms20051243 (PMC6429369; doi:10.3390/ijms20051243)
Supplement: Supplementary file 1 [file ijms-20-01243-s001.pdf]

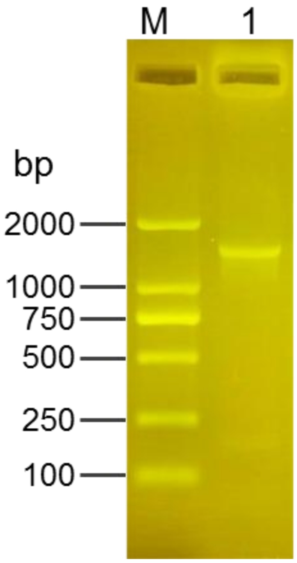

Figure S1. Agarose electrophoresis of the amplified SnHex DNA fragment. M: DNA ladder, 1: PCR product of SnHex (theoretical length is 1461bp).

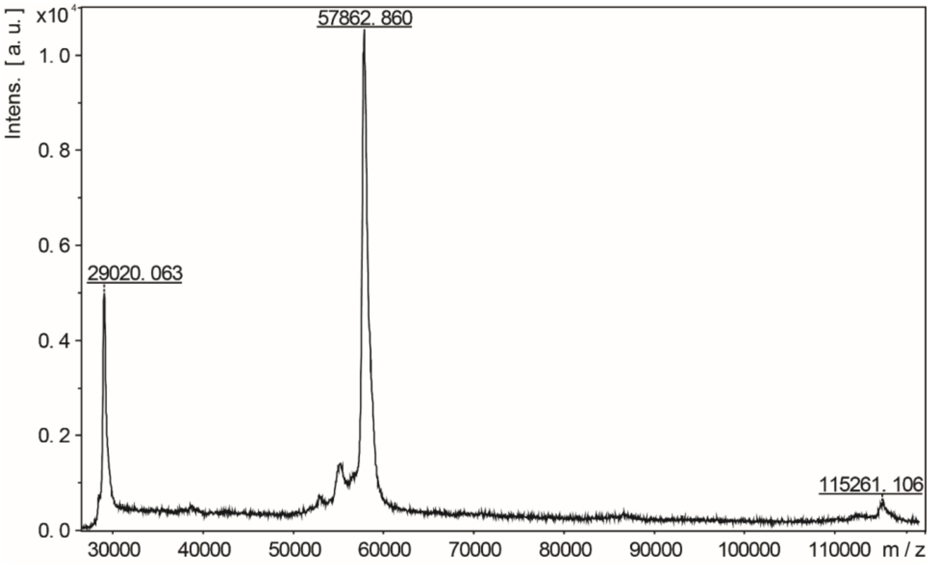

Figure S2. MALDI-TOF analysis of a purified SnHex enzyme preparation.

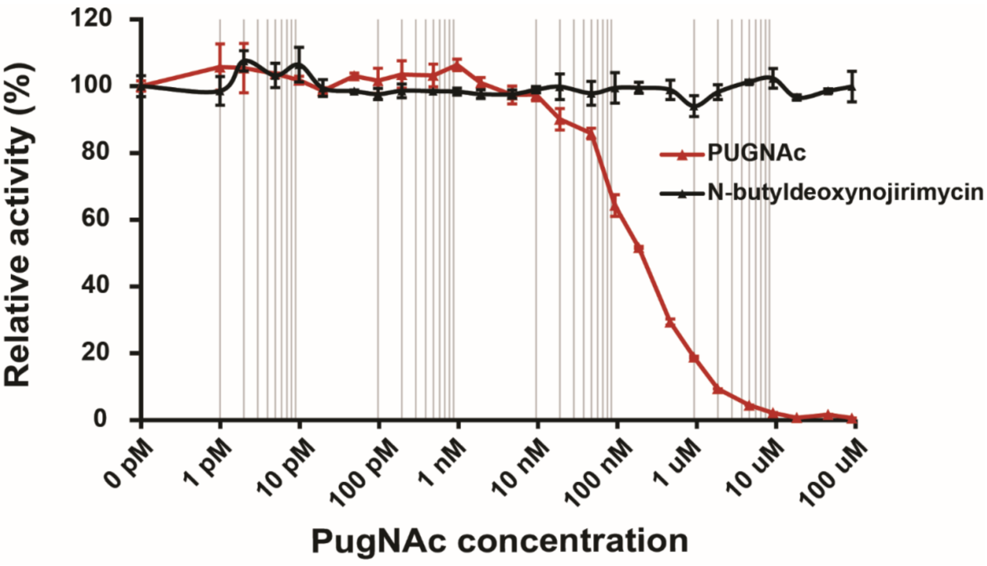

Figure S3. Inhibition of the specific hexosaminidase inhibitor PugNAc

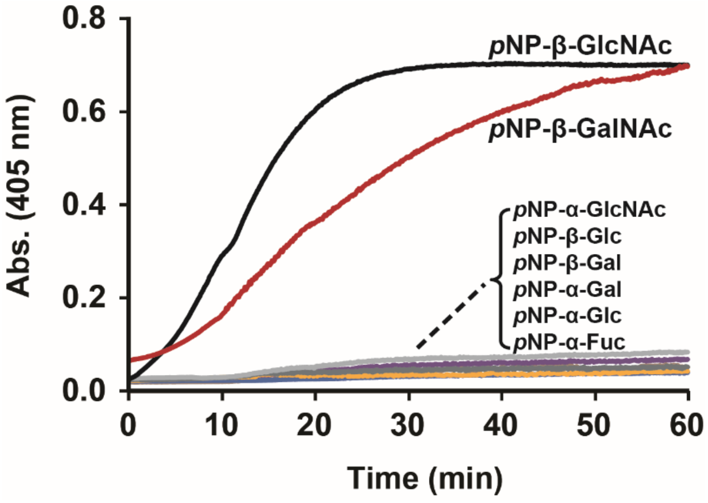

Figure S4. Substrate specificity of recombinant SnHex.

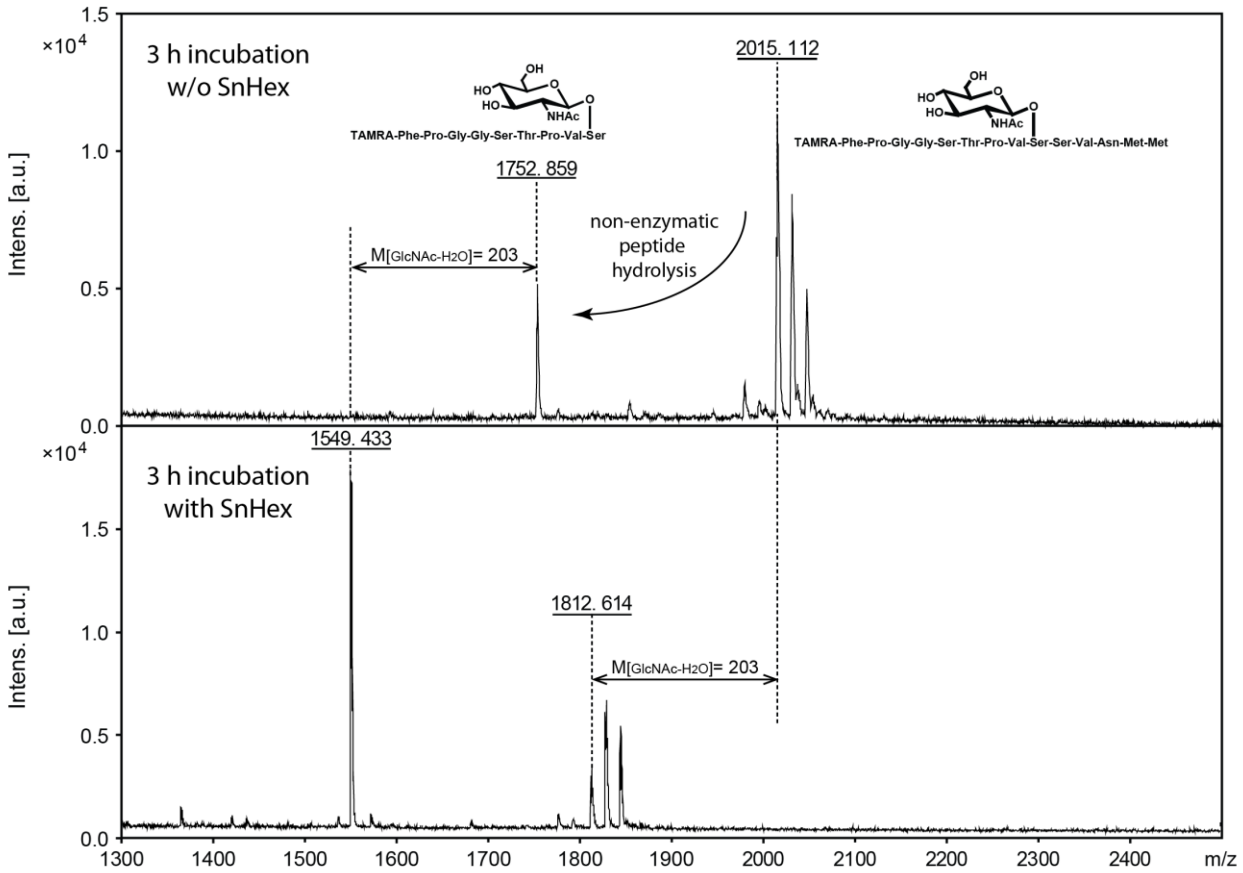

Figure S5. MALDI-TOF mass spectrometric detection of the activity of SnHex on O-glycopeptides. The release of the GlcNAc moiety could be observed from both the intact substrate and the hydrolyzed fragment of the TAMRA-glycopeptide.

```
D3Q4E7 Sn  -------------------------------------------------------------------------------------
P06865 Hs  -------------------------------------------------------------------------------------
B2UP57 Am  -------------------------------------------------------------------------------------
Q54SC9 Dd  -------------------------------------------------------------------------------------
P96155 Vf  MNYRIDFAVLSEHPQFCRFGLTLHNLSDQDLKAWSLHFTIDRYIQPDSISHSQIHQVGSFCSLTPEQDVINSNSHFYCEFSIKTA

             1                                                                              57
D3Q4E7 Sn  ------------------------MRLLRIPTACVILATA-LFIPGSAVADPSHAKAATPSTIPALGE-WTAASGT--YTYSESS
P06865 Hs  ------------------------MTSSRLWFSLLL-AAA--------FAGRATALWPWPQNFQTSDQRYVLYPNNFQFQYDVSS
B2UP57 Am  -----------------------------MARPLPILGGILLSFSPPAEATAQYSIIPEPSRTEL--------------------
Q54SC9 Dd  ----------------MIN----KFLTIFLIFSIVI-IKV-LS-Q-S-SNEQPLNVVPYPQEVTMIGCNIPLSVGSI------SI
P96155 Vf  PFPFHYYTDGIKAAFVQINDVEPRVRHDVIVTPIALASPYRERSEIPATDAATLSLLPKPNHIERLDGEFALTAGSQ-------I

             58                                                                            131
D3Q4E7 Sn  RLVVDPDHADELTDEAQTAAADLAALTGTEPE------VVTGEPETGDVSLALGGGEPL----GAEGYSLDV-GDTFAITANADA
P06865 Hs  AAQPG----CSVLDEAFQRYRDLLFGSGSWPRPYLTGKRHTLEKNVLVVSVVTPGCNQLPTLESVENYTLTINDDQCLLLSETVW
B2UP57 Am  -------------------------------RQETAK-TLQ---------LL--SDQEVPTL-ETDAYRLTVTPQGAHLASGGRE
Q54SC9 Dd  KSNIE----STILSISISRYQSLFFPF-------VSNNVLKDSSSNIELSLIIASDDETLELGIDESYFLLVNQDTYQIKANTIY
P96155 Vf  SLQSS----CA-------------ETAATWLKQELTH-LYQWQPHDIGSADI--VLRTNPTL-DEGAYLLSVDRKPIRLEASSHI

             132                                                                           211
D3Q4E7 Sn  GAFYGTRTLLQLVKQ-----DKQIPAGTATDVPKKPERGLMVDVGRKYMSVEWLKKHIKDLSYAKLNYFHLHLSDTFGFRLESET
P06865 Hs  GALRGLETFSQLV-WKSAEGTFFINKTEIEDFPRFPHRGLLLDTSRHYLPLSSILDTLDVMAYNKLNVFHWHLVDDPSFPYESFT
B2UP57 Am  GRIYGLATLRQLRDQ-LAGQPEGIPCGVITDKPRYPWRGLMVDPARHFIPAADLKKFVDMMAYYKFNRLHLHLTDNQGWRLPVPG
Q54SC9 Dd  GAMRGLETFKQMVVYDVVENSYSLTCAEVVDYPTYQWRGLLVDNARHFLPKNMVLHIIDSMGYNKFNTMHWHLIDTVAFPVESKT
P96155 Vf  GFVHASATLLQLV-R-PDGDNLLVPHIVIKDAPRFKYRGMMLDCARHFHPLERVKRLINQLAHYKFNTFHWHLTDDEGWRIEIKS

             212                                                                           270
D3Q4E7 Sn  HPEVTSED----------------------HYTKAEIRELVDLAAKYHVTLVPEIDVPGHVNAMLAQHPELQLEDQSGNK----D
P06865 Hs  FPELMRKGSYNP---------------VTHIYTAQDVKEVIEYARLRGIRVLAEFDTPGHTLSWGPGIPGLLTPCYSGSE----P
B2UP57 Am  YPKLKSVASRREES------F-GDGIPHEGMYTKQELKELVAYCAARGIDVIPEIDMPGHNQALHAAYPEFFCFPKPDMNVRTTA
Q54SC9 Dd  YPKLTEAL--LG---------------PGAIITHDDILEVVAYAKTYGIRVIPEFDVPGHSASWGVGYPELLSNCPGYPQ----S
P96155 Vf  LPQLTDIGAWRGVDEVLEPQYSLLTEKHGGFYTQEEIREVIAYAAERGITVIPEIDIPGHSRAAIKALPEWLFDEDDQSQYRSIQ

             271                                                                           350
D3Q4E7 Sn  PY---FLDLSNPDAYTLVEDLIKEYLPLFPGPYWHIGADEYVGDYG-KYPQLEAYA-KEHYGPDAVGKDAYYGFINWANEIVRDG
P06865 Hs  SGTFGPVNPSLNNTYEFMSTFFLEVSSVFPDFYLHLGGDEVDFTCWKSNPEIQDFMRKKGFGEDFK--QLESFYIQTLLDIVSSY
B2UP57 Am  GNSKELVCPQKPEVWKFYASVFNELKDIFPSGIVHLGGDEAPTELWEKCPLCREARTRAAMKDEQE---QMKAFFAKTAALLAKN
Q54SC9 Dd  SI---PLDCSNPYTYSFLENFFSEIAPLFQDSYFHTGGDELVIDCWANDTSIQKWMKTNNYNTSD----AFQYFEDQLDVILKSI
P96155 Vf  YYNDNVLSPALPGTYRFLDCVLEEVAALFPSHFIHIGADEVPDGVWVNSPKCQALMAEEGYTDAKE---LQGHLLRYAEKKLKSL

             351                                                                           429
D3Q4E7 Sn  GKTTRMWNDGIKEGDGTIAPEANIVVDYWSTHG-ID----PQKLLDRGH-TVMNASWTPTYYVLGGTDIDRRWLYEDWNPDLFEK
P06865 Hs  GKGYVVWQEVFDNKVK-IQPDT-I-IQVWREDIPVNYMKELELVTKAGFRALLSAPW----YLNRI----------SYGPDWK--
B2UP57 Am  GQTPQFWYEG-NAGIY--HPGET--VYAWRQGQAL---QSIEKTKKAGLNLIMASSE--YCYLDFPQIQG--QRNWGWMKTTTLQ
Q54SC9 Dd  NRTKIAWNDVLQHGVK-FDKETTL-VQTWTN------INDLRDVLAAGYKTITSFFF----YLDRQSPTGNHYH-YEWQDTWE--
P96155 Vf  GKRMVGWEEA-QHGDK-VSKDTV--IYSWLSEQAA---L---NCARQGFDVILQPGQ--FTYLDIAQDYAPEEPGVDWAGVTPLE

             430                                                                           490
D3Q4E7 Sn  TLTIDDPK-----------RNLGSKIHVWCDHPDAQTEDQITEGIRLPLRGLAQQTWGSPKLVE---GYDDFV--------AI--
P06865 Hs  DFYIVEPLAFEGT-PEQKALVIGGEACMWGEYVDNTNL---VPRLWPRAGAVAERLWSNKLTSDLTFAYERLS--------HFRC
B2UP57 Am  KCYDLDPAFGKPEKEA--GHIRGVHAPVWAERLPDLNH--LLYRAYPRACAIAEAGWSPMGVRSWENFRRKLADHRQFILKRFNY
Q54SC9 Dd  DFYASDPRLNI---TSNAENILGGEATMFGEQVSTVNW---DARVWPRAIGISERLWSATEINNITLALPRIG--------QFSC
P96155 Vf  RAYRYEPLVEVPEHDPLRKRILGIQCALWCELVNNQDR--MDYMIYPRLTALAGSGLDTKIPA----------------------

             491                                 503
D3Q4E7 Sn  ---DDAIG---RAPGFSSL--------------------------
P06865 Hs  ELLRRGVQAQPLNVGFCEQEFEQT---------------------
B2UP57 Am  DMERT-QG------------------NEPAFRWENNK--------
Q54SC9 Dd  DMSRRGISSGPLFPDFCSLPDDLSFSFKPVYQLSKDEIKLILKKK
P96155 Vf  ---------------------------------------------
```

Figure S6. Protein alignment of SnHex and functionally characterized β-*N*-acetylhexosaminidases from *Homo sapiens* (Hs), *Vibrio furnissii* (Vf), *Dictyostelium discoideum* (Dd), *Akkermansia muciniphila* (Am). The Uniprot identifier of each protein is shown at the beginning of each line. The amino acids highlighted in red show the catalytic residues.

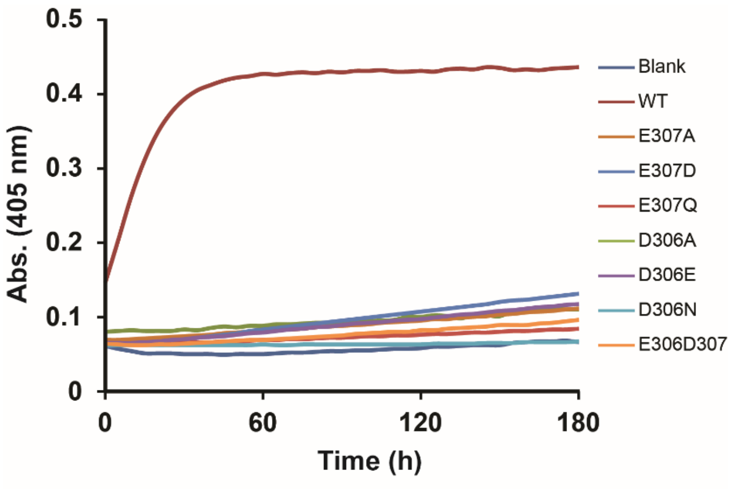

Figure S7. Photometric analysis of the wild-type and mutant variants of SnHex using *p*NP-β-GlcNAc as substrate.

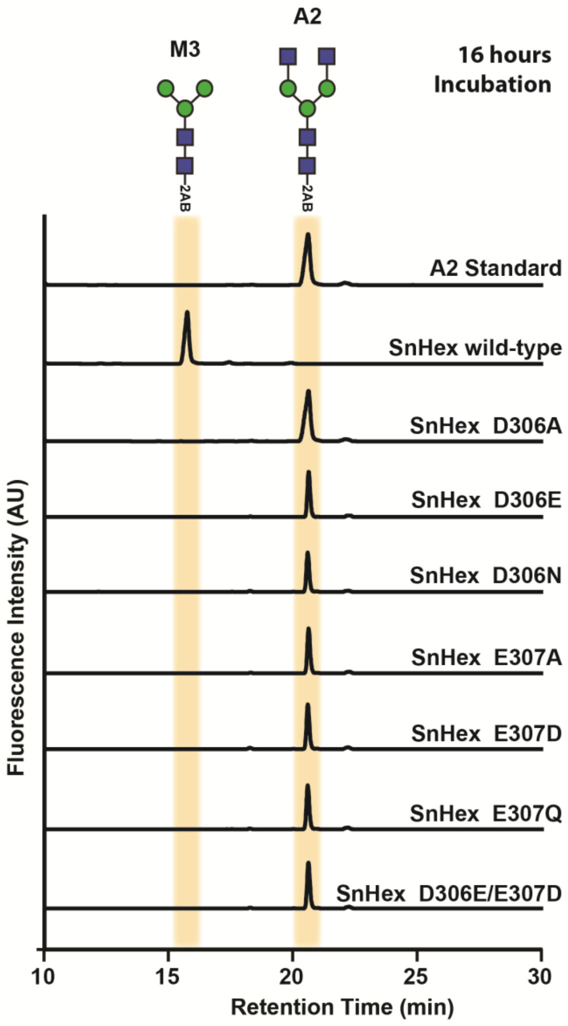

Figure S8. UPLC chromatograms of the wild-type and mutant variants of SnHex using A2 glycan standard as substrate.

```
  1 MVPSSHVRDMTASQDAALAAEWAAGVAYSVGTRVTYQGRLYECRQPHTSQ  50
 51 ADWTPVAVASLWLDLGPAGGGEPDAGSGGTDAGTGGDVTAPTVGLSASAS 100
101 RIIAVGPLSLTATATDDVGVTRVEILENGAVVATGSQFSRAFSGWEQNGT 150
151 YVYAVRAYDAAGNVGTTTLTVVVEIPGGPPPGGKRIVGYFTAWGIYARNY 200
201 HVSNVQPSKLTHINYAFSNISGDGRCILGDPFADIDKSGGWQGEWDPGQL 250
251 RGNFRAFKEMKRQNPHLKLLISVGGWSWSTHFSTVASSPASRAAFVKSCV 300
301 DLYIRGQYPGVDPVNGEGVFDGIDIDWEYPVGGGLPGNSNSPADKQNYTL 350
351 LMQEFRSQLNAVTTQTGKPYLLTIATGASPDLLENKQETKKLSDVLDWIN 400
401 VMSYDYHGAFESTVNFHSALHRVTGDPGAATGFYTDGSVSKMLALGVPPA 450
451 KIVVGVPFYGRGWGSVPNVNNGLFQSGVPTRGTWDDGSSGLTGVFDFKDI 500
501 KANYERPGSGYTKFFHPEAKEAYVYNPATGIWIGYDDVQSINAKADYILN 550
551 KNLGGAMFWELSGDDGSLLDALARKLRLEHHHHHH.               585
```

Figure S9. Translated open reading frame of the MxChi chitinase gene. The cloning and expression of the gene was performed as follows: Genomic DNA was isolated from approximately 50 mg of wet cell pellet obtained from a *Myxococcus xanthus* DK1622 cell culture. The oligonucleotide primers for amplifying the target gene were designed based on the annotated genome data of this organism (sense primer 5'CATATGGTGCCGTCGTCTC ACGTTCG3' and antisense primer 5'CTCGAGGCGCAGCTTCCGTGCCAG3', containing *Nde*I and *Xho*I restriction sites (underlined sequence). Gene amplification, restriction, ligation, transformation, expression and purification were performed as described in the same manner as described for the SnHex gene. The protein concentration was determined at 0.45±0.09 mg/mL. It was described that GH18 family chitinases contain a catalytically important DXDXE motif [1]. By introduction of a D348A mutation in MxChi this motif was altered from DXDXE into DXAXE. The catalytically inactive MxChi mutant variant D346A was generated using the same site-directed mutagenesis procedure described for generating the SnHex mutants using the primers TTCGACGGCATCGACATCGCCTGGGA GTACCCGGTCGGCG and CGCCGACCGGGTACTCCCAGGCGATGTCGATGCCGTCGAA.

Table S1. Effect of different chemical additives on recombinant SnHex.

Chemical compounds	Relative activity (%)
Blank	100±0.3
0.1 M Urea	81±2.0
0.5 M Urea	72±2.2
1 M Urea	56±0.4
0.1% SDS	53±1.0
0.5% SDS	52±3.0
1% SDS	42±0.4
1 mM 2ME	92±1.3
10 mM 2ME	100±1.4
50 mM 2ME	84±2.0
0.1% Triton X-100	97±1.2
0.5% Triton X-100	94±1.1
1% Triton X-100	105±0.8
1mM Iodoacetamide	110±4.3
5 mM Iodoacetamide	108±0.9
10 mM Iodoacetamide	107±2.2
1 mM Ethylmaleimide	61±1.7
5 mM Ethylmaleimide	60±2.7
10 mM Ethylmaleimide	54±1.7

Table S2. Detailed annotation of N-glycan structures

Annotation	Detailed N-glycan depiction
M3	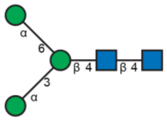
M5	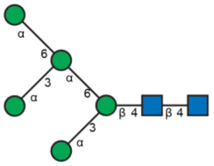
A1	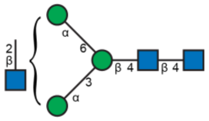
A2	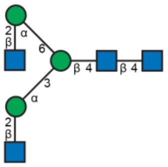
A3	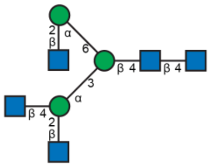
A4	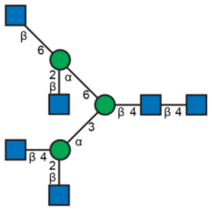
M5A1B	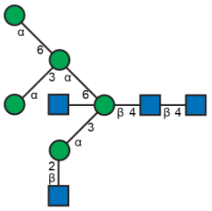
F6A2B	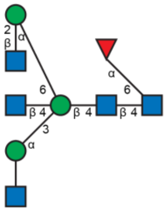

Table S3. Primers used in directed-site mutation for SnHex.

Primer	Sequence (5′-3′)
D306A	F: CGTACTGGCACATCGGCGCCGCAGAATACGTCGGCGACTACG<br>R: CGTAGTCGCCGACGTATTCTGCGGCGCCGATGTGCCAGTACG
D306E	F: CGTACTGGCACATCGGCGCCGAAGAATACGTCGGCGACTACG<br>R: CGTAGTCGCCGACGTATTCTTCGGCGCCGATGTGCCAGTACG
D306N	F: CGTACTGGCACATCGGCGCCAATGAATACGTCGGCGACTACG<br>R: CGTAGTCGCCGACGTATTCATTGGCGCCGATGTGCCAGTACG
E307A	F: CTGGCACATCGGCGCCGATGCTTACGTCGGCGACTACGGG<br>R: CCCGTAGTCGCCGACGTAAGCATCGGCGCCGATGTGCCAG
E307D	F: CTGGCACATCGGCGCCGATGATTACGTCGGCGACTACGGG<br>R: CCCGTAGTCGCCGACGTAATCATCGGCGCCGATGTGCCAG
E307Q	F: CTGGCACATCGGCGCCGATCAATACGTCGGCGACTACGGG<br>R: CCCGTAGTCGCCGACGTATTGATCGGCGCCGATGTGCCAG
D306E/E307D	F: CGTACTGGCACATCGGCGCCGAAGATTACGTCGGCGACTACGGG<br>R: CCCGTAGTCGCCGACGTAATCTTCGGCGCCGATGTGCCAGTACG

References:

[1] Vaaje-Kolstad, G.; Houston, D. R.; Rao, F. V.; Peter, M. G.; Synstad, B.; van Aalten, D. M.; Eijsink, V. G.; Structure of the D142N mutant of the family 18 chitinase ChiB from *Serratia marcescens* and its complex with allosamidin. Biochimica et biophysica acta 2004, 1696,103-11.
